# Database of Nonaqueous Proton-Conducting Materials

**DOI:** 10.1021/acsami.4c22618

**Published:** 2025-03-10

**Authors:** Harrison J. Cassady, Emeline Martin, Yifan Liu, Debjyoti Bhattacharya, Maria F. Rochow, Brock A. Dyer, Wesley F. Reinhart, Valentino R. Cooper, Michael A. Hickner

**Affiliations:** † Department of Chemical Engineering and Materials Science, Michigan State University, East Lansing, Michigan 48824-1312, United States; ‡ Energy Technologies Area, Lawrence Berkeley National Laboratory, Berkeley 94720-8099, California, United States; § Department of Chemical Engineering, University of Michigan, Ann Arbor, Michigan 48109-1382, United States; ∥ Materials Science and Technology Division, Oak Ridge National Laboratory, Oak Ridge, Tennessee 37831-2008, United States; ⊥ Materials Science and Engineering, The Pennsylvania State University, University Park, Pennsylvania 16802, United States; # Department of Physics and Astronomy, Ursinus College, Collegeville, Pennsylvania 19426, United States; ∇ Institute for Computational and Data Sciences, The Pennsylvania State University, University Park, Pennsylvania 16802, United States

**Keywords:** small molecules, proton conductivity, Python, database, nonaqueous molecules, imidazole, acid-doped, proton exchange membrane

## Abstract

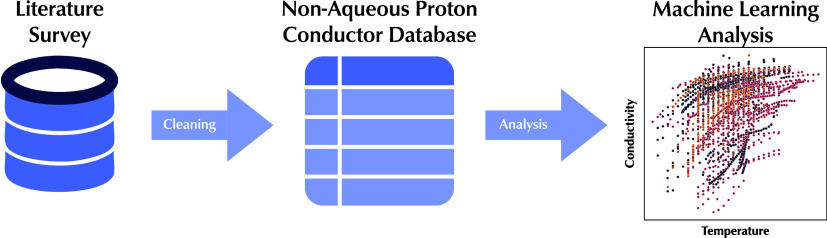

This work presents the assembly of 48 papers, representing 74 different compounds and blends, into a machine-readable database of nonaqueous proton-conducting materials. SMILES was used to encode the chemical structures of the molecules, and we tabulated the reported proton conductivity, proton diffusion coefficient, and material composition for a total of 3152 data points. The data spans a broad range of temperatures ranging from −70 to 260 °C. To explore this landscape of nonaqueous proton conductors, DFT was used to calculate the proton affinity of 18 unique proton carriers. The results were then compared to the activation energy derived from fitting experimental data to the Arrhenius equation. It was found that while the widely recognized positive correlation between the activation energy and proton affinity may hold among closely related molecules, this correlation does not necessarily apply across a broader range of molecules. This work serves as an example of the potential analyses that can be conducted using literature data combined with emerging research tools in computation and data science to address specific materials design problems.

## 1 Introduction

Developing efficient proton-conducting materials is crucial for advancing new electrochemical technologies, including fuel cells, electrolyzers, redox flow batteries, and sensors.^1−3^ While aqueous proton-conducting systems are often utilized in this role, they are usually limited to operating at temperatures below 100 °C.^4^ This temperature constraint has limited the advancement of proton-conducting technologies in applications where elevated temperatures are essential for improved efficiency or reaction kinetics.^5^ To overcome this challenge, researchers have turned their attention to the development of nonaqueous proton conductors, which offer the potential to operate at higher temperatures than their aqueous counterparts.

The most notable example of nonaqueous proton-conducting materials in fuel cells is the design of phosphoric acid-based membranes.^6,7^ While there are a few well-studied systems, including phosphoric acid and azoles, the design and discovery of new nonaqueous proton-conducting materials remain challenging due to the complex factors affecting proton transport and the limited chemical motifs explored thus far.^8−11^

Further complicating the analysis of these materials, nonaqueous proton-conducting systems often include a dopant in addition to the primary conductor. These dopants typically serve to increase the concentration of hydrogen ions, thereby promoting proton dissociation within the primary material.^12,13^ However, the introduction of dopants increases the complexity of the system, influencing the material’s viscosity and overall conductivity. Therefore, the selection of an appropriate dopant and the optimization of doping ratios are critical considerations in system design.

Machine learning has emerged as a powerful tool for materials design and discovery across various application spaces. However, these methods often require large quantities of experimental data during the training process.^14−17^ There are now several large chemical and material databases, including repositories such as The Materials Project and JARVIS, which primarily contain theoretical data, and others like the Inorganic Crystal Structure Database and the Crystallography Open Database, which may include both experimental and theoretical data.^18−23^ Once a data set is collected, a series of steps including data cleaning, featurization, model training, and visualization can be performed to predict the properties or features of new compounds.^24−27^

To address the need for advanced materials design in nonaqueous proton conductors, we developed a comprehensive database derived from an extensive literature review. This database encompasses information from 48 papers, covering 74 unique materials, including proton-conducting matrices such as phosphoric/phosphonic acid-based materials and azole-based proton carriers. A key feature of the database is the quantification of doping levels and proton-donating dopants. To provide further insights, we performed density functional theory (DFT) proton affinity calculations, exploring how this quantity may influence the conductivity of the molecules in the database. By making this database publicly available, we aim to accelerate the discovery and development of high-performance, nonaqueous proton conductors, ultimately contributing to advancements in energy conversion and storage technologies.

## 2 Methods

### 2.1 Database Construction

The database was constructed by extracting quantitative data from plots and tables in the previously published academic literature. Relevant papers were identified through Web of Science and Google Scholar searches using keywords related to nonaqueous proton conductors. Additional sources were found by the following references within these papers.

While the primary focus was on extracting proton conductivities, other parameters, such as diffusion coefficients, were also included when available. The study extracted values for both doped and undoped systems.

Molecules were encoded using *Simplified Molecular-Input Line-Entry System* (SMILES), a standardized method for representing molecular structures as text strings.^28^ This encoding allows for the conversion of molecules—typically reported in research papers as diagrams—into a format that can be easily stored in databases and parsed using widely available cheminformatics tools. SMILES strings were generated by recreating the chemical structures in ChemDraw (ver. 23.1.1, Revvity, Waltham, MA) and utilizing the built-in “Copy as SMILES” feature. SMILES strings were verified using RDKit (ver. 2023.03.1) to regenerate the molecules from the generated ChemDraw SMILES.^29^

One limitation of SMILES is its inability to directly encode polymer systems, particularly when it comes to representing repeat units and defining connections between them. Although SMILES is sometimes adapted to describe simple aspects of the polymer structure (e.g., by encoding specific repeat units), this approach lacks the fidelity required for a robust representation of full polymer connectivity and variability. Although efforts have been made to extend the SMILES syntax for polymer representation (e.g., BigSMILES), the tools for working with polymers in cheminformatics are still in their early stages.^30−32^ Consequently, this study focused solely on small-molecule (i.e., nonpolymer) proton conductors. As the BigSMILES notation continues to develop, a follow-up study examining nonaqueous polymeric proton conductors will be considered.

The raw data were organized into an Excel document containing two sheets: *Compounds* and *Parameters*. The *Compounds* sheet stores information about individual molecules, with each entry comprising a unique Compound ID and its corresponding SMILES string. The *Parameters* sheet contains columns that link primary and (where relevant) dopant IDs to the related molecules in the *Compounds* sheet, doping ratios, and extracted experimental parameters. Each entry in the *Parameters* sheet also contains the DOI of the study so that the original data source is easily accessible and searchable. The Excel document containing the raw data is included in the Supporting Information. The database includes results from a total of 48 different papers, with 74 unique proton conductor matrix molecules and 33 dopant molecules.^8,33−79^

Data extraction from plots was performed using Graph Grabber 2.0.2 (Quintessa Ltd., Oxfordshire, U.K.). To maintain the integrity of the raw data, parameters were entered into the database using the original terminology and units employed in each study. This approach ensures fidelity to the source material and prevents potential errors from unit conversions or terminology during the data entry stage. For instance, conductivity measurements can be entered as “log(S/cm)” from one study and “mS cm^–1^” from another, reflecting the diverse reporting conventions in the literature. We preserved the original context of each study, and our approach allows for verification of the raw data against the sources as needed. All unit conversions were performed in the processing and analysis stages of the work.

### 2.2 Data Cleaning and Processing

To facilitate the integration of the database with machine learning algorithms, a data cleanup process was implemented. This process focused on extracting and standardizing two key parameters: the proton conductivity and the proton diffusion coefficient. The raw data for these parameters were converted into a uniform, easily parsable format.

Data cleaning involved five steps: (1) Merging the Compounds and Parameters tables to create a unified data set. (2) Validating SMILES strings using the RDKit Python package and converting them to RDKit canonical SMILES to ensure consistency across all SMILES strings. (3) Develop translation dictionaries to standardize terminology across different papers (e.g., mapping “Proton Conductivity” and “DC Conductivity” to “Conductivity”) since only papers on proton-conducting materials were analyzed. (4) Reversing any transformations applied to the parameters in the original studies (e.g., converting conductivities reported as log (σ) back to σ, and temperatures reported as 1000/*T* back to *T*). (5) Converting all parameters to base SI units.

Following the data cleaning process, the results were stored in two tab-separated value files (one each for conductivity and diffusion coefficients) for easy parsing with machine learning algorithms. Both files are included in the Supporting Information. These two cleaned files, along with the raw data excel document, are also available for download in a Zenodo repository.^80^

#### 2.2.1 Proton Affinity Calculations

DFT was used to compute the proton affinity for a given molecule. Each molecule is represented in the database as a SMILES string in the uncharged (nonprotonated) form. An RDKit script was developed to identify all possible protonation sites within each uncharged SMILES string, generating the corresponding charged SMILES strings. The charged and uncharged SMILES strings were then converted into .xyz files for analysis with DFT.

The DFT calculations in this study were performed using the Quantum ESPRESSO (QE, ver. 6.3) simulation software.^81^ All calculations employed a plane-wave basis set with a kinetic energy cutoff of 100 Ry and an energy convergence threshold of 10^–8^ for self-consistency. Exchange-correlation effects were treated using the vdW-DF-C09 functional, and electron–ion interactions were described by the GBRV ultrasoft pseudopotentials.^82,83^ Brillouin zone sampling was restricted to the Γ-point. For charged (protonated) systems, a positive net charge was explicitly included in the calculations. To minimize spurious interactions between periodic images, all calculations were conducted in the gas phase using a vacuum box with a minimum separation of 16 Å between adjacent molecules. Proton affinity was calculated as the energy difference between the optimized geometries of the neutral and protonated species. Mathematically, the proton affinity (*E*_PA_) can be expressed as eq 1
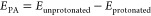
1

## 3 Results and Discussion

**Figure 1:**
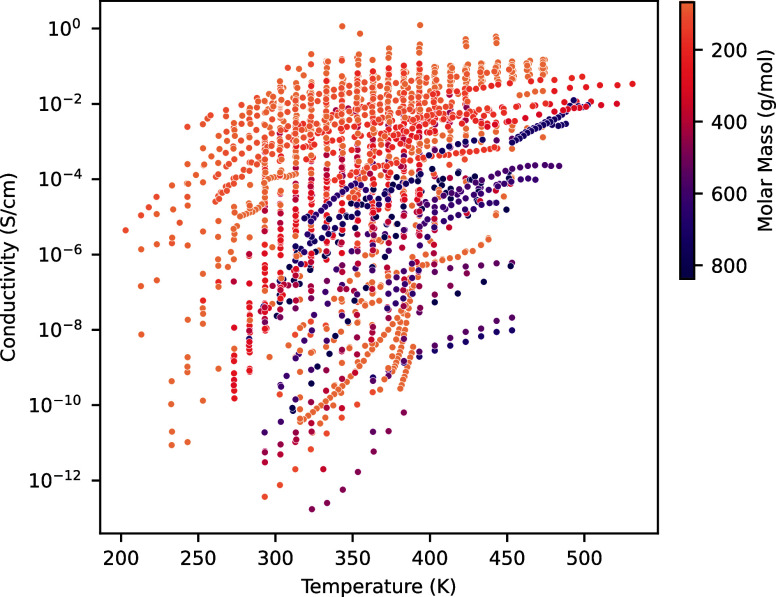
Conductivity as a function of temperature for all available points in the database. The hue represents the molecular weight of the reported molecule.

To illustrate the range of values included in the database and demonstrate the utility of the database, several summary plots have been generated. Figure 1 shows the proton conductivity as a function of temperature with the color representing the molar mass of the conducting molecule.

Molecules with higher molecular weights in Figure 1 tend to have a lower conductivity, although that is not always the case. Conversely, nearly all of the highest conductivity molecules fall within the lower range of molecular weights. This trend is expected as larger molecules are often associated with slower dynamics; however, factors like viscosity or electronic structure can offset this effect. In the case of the best proton conductors, the molecular size is optimized alongside these factors, resulting in smaller molecules delivering superior performance.

Several studies incorporate an additional molecule, termed a dopant, to enhance conductivity. For example, small amounts of the dopant phosphoric acid can be added to imidazole to provide a proton source for conduction. In this system, imidazole is the primary conductor, and the presence of a dopant serves to enhance the conductivity. However, in many cases, the amount of dopant added is so substantial that it exceeds what would typically be considered a modification of the original system. To address this, a third category is introduced, termed “composite,” which applies to systems where the doping level is sufficiently high for the material to be regarded as a composite of two materials rather than a doped system.

**Figure 2:**
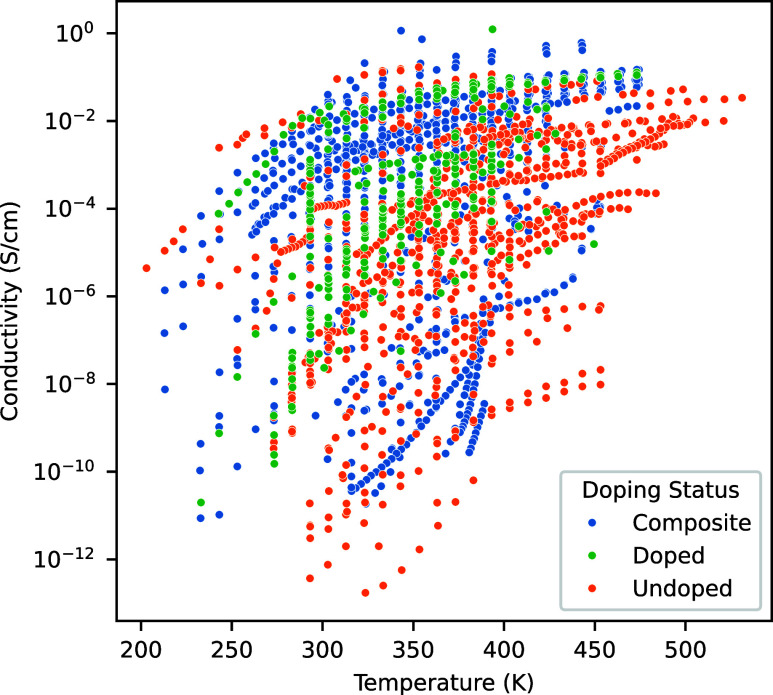
Conductivity as a function of the temperature for all species in the database. The orange points represent data points where a dopant was not used, the green points represent doped systems where the secondary compound is less than 20%, and the blue points represent composite systems where the secondary component is greater than 20%.

Figure 2 presents the conductivity versus temperature data, distinguishing among doped systems, composite systems, and undoped systems. Systems with a doping percentage below 20% are classified as doped, while those with a percentage above 20% are classified as a composite system. The figure demonstrates that, with few exceptions, high conductivities are predominantly observed in systems utilizing a dopant of some kind and that composite systems tend to perform slightly better than systems with lower levels of dopant.

**Figure 3:**
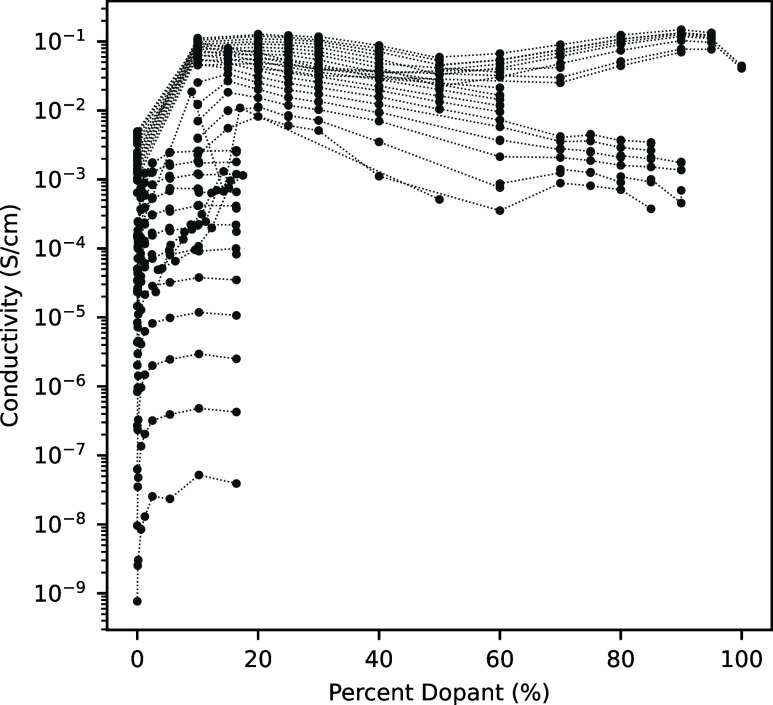
Conductivity as a function of dopant percent for all doped systems in the database. A single set is taken to be a unique molecule-dopant pair at a single temperature. Sets are connected with a thin dotted line to aid the eye in tracking the trend of each set.

To further investigate the effect of doping on the proton conductivity, Figure 3 takes the doped systems and plots the conductivity versus percent dopant for a given temperature. The relationship between conductivity and the dopant is complex, with many factors such as viscosity and interactions between the conductor and the dopant leading to variability. However, Figure 3 does show that for most doped systems, there is an optimum doping level after which conductivity performance levels off or decreases. This optimum dopant concentration has been noted in most individual studies, but Figure 3 highlights the commonality of this doping phenomenon across a wide range of molecule types and absolute values of conductivity.^84,85^

**Figure 4:**
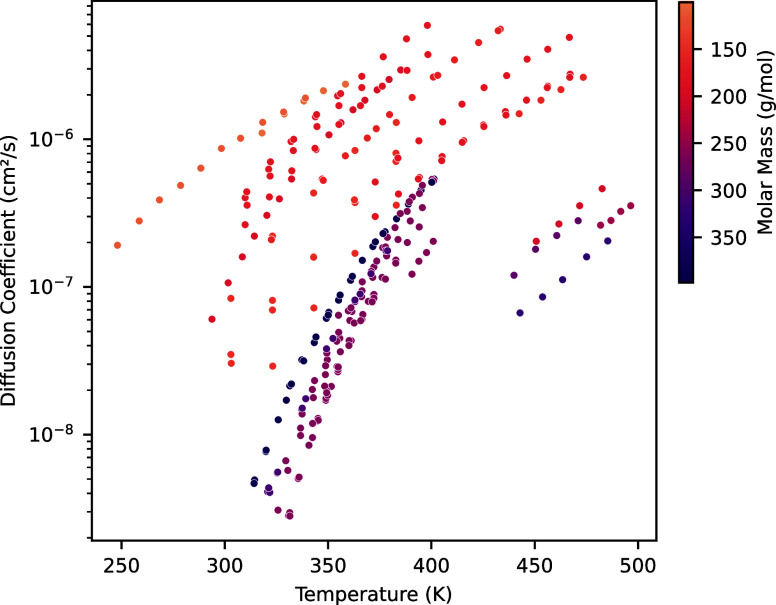
Diffusion coefficient as a function of temperature for all available points in the database. The color hue represents the molecular weight of the reported molecule.

Figure 4 shows the proton diffusion coefficients collected in the database as a function of the temperature. Although fewer studies reported the diffusion coefficients of their molecules compared to conductivity data (the proton diffusion coefficients were extracted or measured using several different methods), the available information reveals trends similar to those observed in the conductivity measurements. Specifically, lower-molecular-weight molecules exhibit higher diffusion coefficients than higher molecular weight compounds. Additionally, as expected, diffusion coefficients increase with temperature.

One limitation of the current database described in this paper is the absence of information regarding the specific methods used to measure the various parameters. This potentially impacts the comparability of values across different studies as variations in measurement techniques may influence the results. However, this limitation is not expected to significantly impede the intended use of the data set as the primary goal is to employ machine learning techniques to identify broad trends within the data. In this context, the differences arising from various measurement methods will average out, allowing for the extraction of meaningful relationships across the data set to predict new molecules. As our data-driven approach is employed more frequently to quantitatively evaluate large data sets, tags for different techniques can be applied to the data to establish further granularity.

### 3.1 Using the Database

To illustrate the practical use of the database, we conducted a small-scale analysis using a set of 18 molecules selected from the database. These molecules were chosen to represent a wide range of molecular weights and structural families of proton carriers. Structural diagrams of the selected molecules are provided in the Supporting Information.

To relate the computed proton affinity to the experimental conductivity, the Arrhenius equation was used:
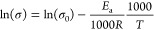
2where σ is the ion conductivity, σ_0_ is the exponential prefactor, *E*_a_ is the activation energy, *R* is the ideal gas constant, and *T* is the temperature.

After the mathematical transformations required in eq 2, a linear regression was used to determine σ_0_ and *E*_a_ for each of the selected molecules. Although studies have shown that the activation energy can have some temperature dependency, all selected molecules had a high degree of linearity indicating that the Arrhenius equation is valid in this situation.^86^ Plots of the conductivity versus temperature, along with the Arrhenius fits, are included in the Supporting Information.

**Figure 5:**
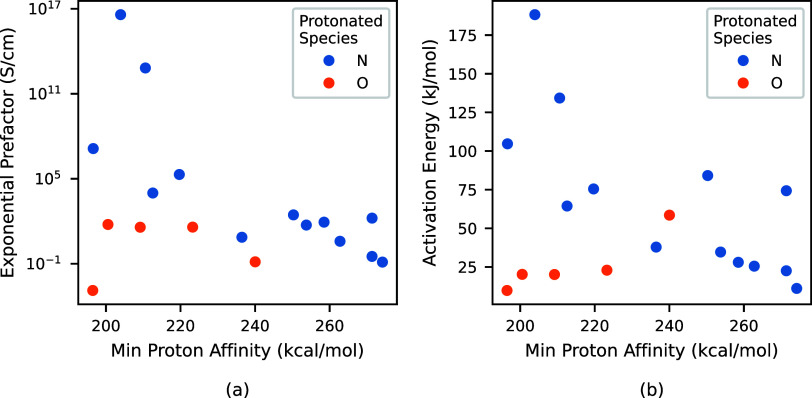
(a) Exponential prefactor and (b) activation energy for 18 molecules selected from the database as a function of minimum proton affinity. The proton affinity is the minimum proton affinity of all available states for a given molecule. Colors represent the atom within the molecule that was protonated to produce the protonated molecule.

Figure 5 shows (a) the exponential prefactor and (b) the activation energy as a function of DFT-computed proton affinities. The proton affinity reported in Figure 5 is the minimum proton affinity of all of the available protonated states for each molecule. This is based on the idea that the most minimally bound site will dominate the overall proton transfer kinetics as it offers the lowest energy barrier for proton dissociation.^87^ It is also possible that the maximum proton affinity plays a part of the conductivity dynamics as it may play a role in trapping protons, and a plot of the exponential prefactors and activation energies versus maximum proton affinity is included in the Supporting Information.

The points in Figure 5 are colored according to whether the protonated site is a nitrogen or oxygen atom, grouping them into two categories: azoles (nitrogen) and phosphonic or carboxylic acids (oxygen).

It is generally anticipated that activation energy and proton affinity will exhibit a positive correlation, with molecules possessing higher proton affinities expected to have correspondingly higher activation energies.^88−90^ While this trend holds in Figure 5 for molecules where oxygen is protonated, an inverse relationship is observed for molecules in which nitrogen is protonated, which was not expected.

While this finding does not invalidate our previous understanding of the relationship between proton affinity and conductivity, it suggests that additional factors may play a significant role in addition to proton affinity in determining the proton conductivity of a system. Examining proton affinity correlations with proton conductivity likely holds within specific classes or groups of molecules but may not extend across broader regions in the latent space. Identifying the appropriate classification and categorization of molecules to preserve these relationships and potentially uncover other factors influencing proton conductivity is an area where machine learning can enhance our understanding.

Additionally, in a broad survey of different experimental works, there may be confounding factors that skew the trends observed in the data above. Details of sample purity, conductivity, electrode surfaces, and changes in viscosity with doping influence these types of measurements. Furthermore, the database includes data from both liquid and solid phases with some conductivity discontinuities attributable to phase transitions. While it is not feasible to account for every detail, it is expected that as more data are added to the database, these confounding factors may be smoothed out.

## 4 Conclusions

A database of nonaqueous proton-conducting small organic molecules has been constructed containing the conductivity and, where available, diffusion coefficients for 74 distinct molecules. The database captures these parameters across multiple temperatures (from −70 to 260 °C), both with and without dopants, and at various doping ratios. In total, the database contains 3152 individual data points from 48 sources, providing a rich resource for machine learning analysis in the development of new proton-conducting materials.

The dataset is presented in both raw and cleaned forms and is available in easily parsed tab-separated value files. This format will facilitate the integration of the dataset into machine learning models for designing new nonaqueous proton-conducting molecules.

Initial analysis of the data set revealed that the reported conductivities ranged over 12 orders of magnitude. Notably, molecules with lower molecular weights and doped systems tended to exhibit the highest conductivities.

An analysis of a sample subset of molecules from the database revealed that a higher proton conductivity of a material does not always correlate with a lower proton affinity calculated by using DFT. The ongoing work aims to develop models that can identify the conditions under which the proton affinity-conductivity relationship holds and determines additional descriptors that can guide the design of new proton-conducting materials.

As methods for representing polymers in machine learning models become more robust and widespread, this database could be expanded to include polymer-based nonaqueous proton conductors.

Future studies will use machine learning, regression analysis, and simulations to develop models for predicting the proton conductivity of small molecules using the database as a source of training data. The dataset could also be used to examine the degree to which different research groups’ results differ when performing measurements on the same compounds. Because each study is encoded with a DOI, a researcher could use a DOI API to examine the author list and sort the results by the research group. By comparing the reported values of proton conductivity and diffusion coefficients for identical compounds across different studies, it would be possible to assess the reproducibility and consistency of the measurements. Combining these tools with methods for generating new molecules will aid the development and understanding of new nonaqueous proton-conducting models.

## Data Availability

The database files developed and used in this study are publicly available on Zenodo.^80^

## Supporting Information Available

Cleaned database files in *.tsv format and raw database in *.xlsx format (ZIP)
Chemical diagrams and arrhenius fits of selected molecules and plots of exponential prefactor and activation energy as a function of maximum proton affinity (PDF)
