# Supplementary material for: Database of Nonaqueous Proton-Conducting Materials
Source: ACS Appl Mater Interfaces. 2025 Mar 10;17(11):16901–8. doi: 10.1021/acsami.4c22618 (PMC11931497; doi:10.1021/acsami.4c22618)
Supplement: Supplementary file 2 — am4c22618_si_002.pdf [file am4c22618_si_002.pdf]

Supporting Information

A database of non-aqueous proton conducting materials

Harrison J. Cassady[a], Emeline Martin[a,f], Yifan Liu[c], Debjyoti Bhattacharya[d], Maria F. Rochow[a], Brock Dyer[f], Wesley F. Reinhart[d,e], Valentino R. Cooper[c], Michael A. Hickner*[,a]

[a]*Department of Chemical Engineering and Materials Science, Michigan State University, East Lansing, MI, USA*
[b]*Current Affiliation: Department of Chemical Engineering, University of Michigan, Ann Arbor, MI, USA*
[c]*Materials Science and Technology Division, Oak Ridge National Laboratory, Oak Ridge, TN, USA*
[d]*Materials Science and Engineering, Penn State, University Park, PA, USA*
[e]*Institute for Computational and Data Sciences, Penn State, University Park, PA, USA*
[f]*Department of Physics, Ursinus College, Collegeville, PA, USA*
**Corresponding Author.*
Email: mhickner@msu.edu

S1. Database Files

The raw data collected from the studies is included in the file:

- `ProtonConductingDatabase-RawData.xlsx`

This Excel file contains two sheets: *Compounds* and *Parameters*. The *Compounds* sheet lists all molecules, which can be used either as conductors or dopants. Each compound is assigned a unique number in the `ID` column, which is used for identification. The `Name` column provides the common name of each compound as referenced in the literature. This column is included for convenience and is primarily intended for manual inspection. The `SMILES` column represents the molecular structure as a SMILES string.

The *Parameters* sheet contains the raw data extracted from the studies. The `ID` and `Dopant ID` columns correspond to the unique IDs of the molecules, linked to the `ID` column in the *Compounds* sheet. To allow the columns to be parsed as an integer, a value of -1 is used to indicate the absence of a molecule.

The majority of parameters entered into the database are proton conductivities and diffusion coefficients. To facilitate parsing these parameters with machine learning tools, the raw data was cleaned and is provided in two tab-delimited files:

- `CleanedConductivityDatabase.tsv`
- `CleanedDiffusionDatabase.tsv`

In these cleaned files, the raw data has been standardized into a consistent format, with conductivity, diffusion coefficient, and temperature expressed in SI base units. While the compound IDs have been preserved, the two sheets from the raw data have been merged so that the SMILES representation of each molecule is included with each data point.

S2. Arrhenius Fitting

A subset of 19 molecules was selected from the database for further analysis. The molecular structure of the selected molecules as included in the database is shown in Figure S1.

To determine the activation energy, the conductivity versus temperature data for the selected molecules was fit using the Arrhenius equation:

$$\ln(\sigma) = \ln(\sigma_0) - \left(\frac{E_a}{RT}\right) \tag{S1}$$

The fit for each selected molecule is shown in Figure S2.

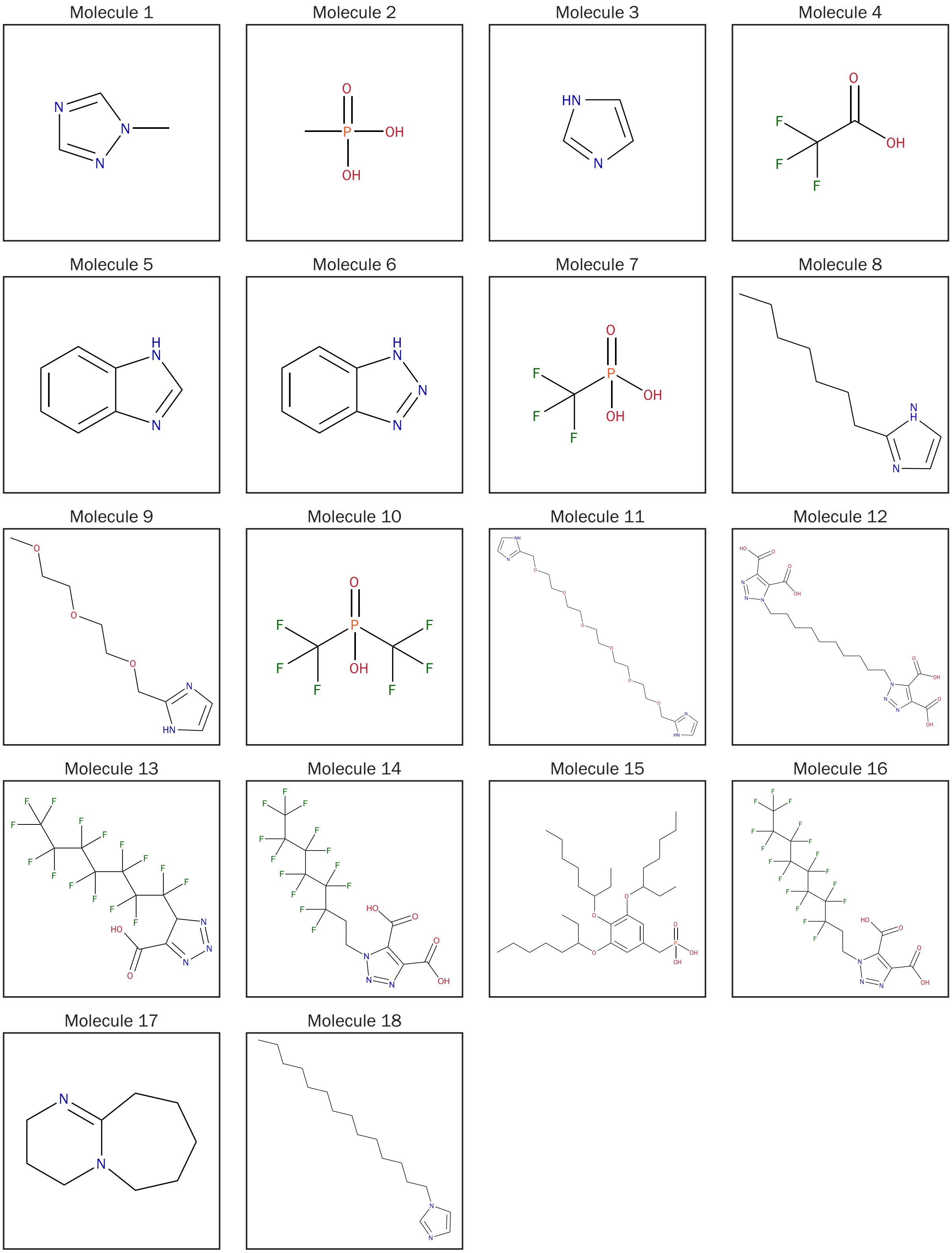

Figure S1: Subset of molecules selected from the database for further analysis.

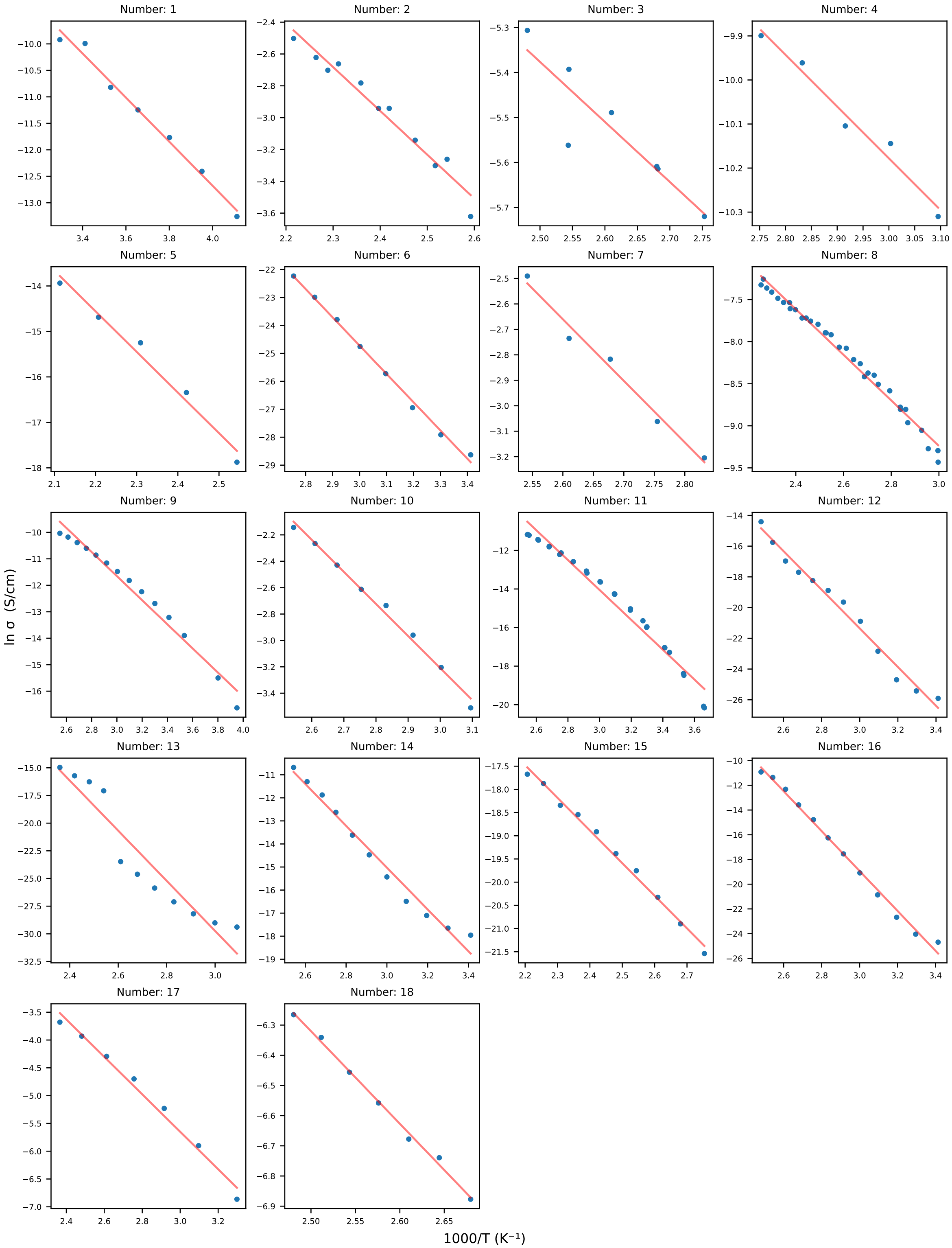

Figure S2: Arrhenius fit for the selected molecules.

S3. Additional Plots

While the manuscript presumed that the minimum proton affinity is the best descriptor for describing the proton conductivity, there are other descriptors that could be useful. Figure S3 presents the Arrhenius fit parameters as a function of maximum proton affinity. The general conclusion from this plot is the same as in the manuscript—that proton affinity alone does not do a good job at predicting the proton conductivity.

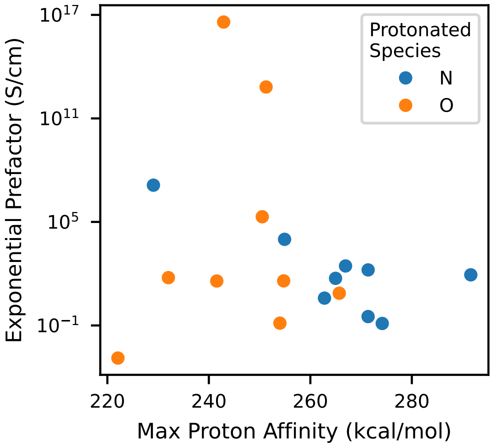

(a)

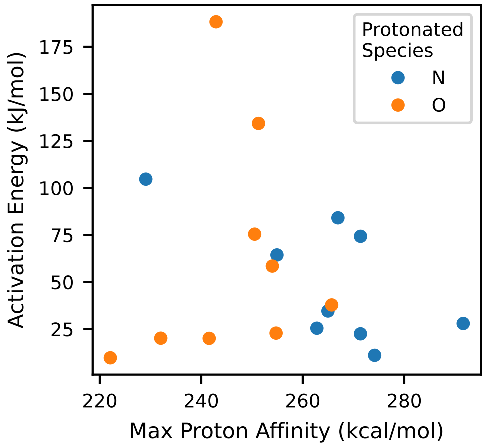

(b)

Figure S3: The (a) exponential prefactor and (b) activation energy for 19 molecules selected from the database as a function of maximum proton affinity. The proton affinity is the maximum proton affinity of all available states for a given molecule. Colors represent the atom within the molecule that was protonated to produce the protonated molecule.
